# Aesculetin Inhibits Airway Thickening and Mucus Overproduction Induced by Urban Particulate Matter through Blocking Inflammation and Oxidative Stress Involving TLR4 and EGFR

**DOI:** 10.3390/antiox10030494

**Published:** 2021-03-22

**Authors:** Su-Yeon Oh, Yun-Ho Kim, Min-Kyung Kang, Eun-Jung Lee, Dong-Yeon Kim, Hyeongjoo Oh, Soo-Il Kim, Woojin Na, Il-Jun Kang, Young-Hee Kang

**Affiliations:** Department of Food and Nutrition and Korean Institute of Nutrition, Hallym University, Chuncheon 24252, Korea; suy0411@naver.com (S.-Y.O.); royalskim@hallym.ac.kr (Y.-H.K.); mitholy@hallym.ac.kr (M.-K.K.); reydmswjd@naver.com (E.-J.L.); ehddus3290@naver.com (D.-Y.K.); ohhyeongju@gmail.com (H.O.); ky4850@naver.com (S.-I.K.); nsm0729@hanmail.net (W.N.); ijkang@hallym.ac.kr (I.-J.K.)

**Keywords:** urban particulate matter, airway thickening, mucus hypersecretion, aesculetin inflammation, oxidative stress

## Abstract

Particulate matter (PM) is a mixture of solid and liquid air pollutant particles suspended in the air, varying in composition, size, and physical features. PM is the most harmful form of air pollution due to its ability to penetrate deep into the lungs and blood streams, causing diverse respiratory diseases. Aesculetin, a coumarin derivative present in the Sancho tree and chicory, is known to have antioxidant and anti-inflammatory effects in the vascular and immune system. However, its effect on PM-induced airway thickening and mucus hypersecretion is poorly understood. The current study examined whether naturally-occurring aesculetin inhibited airway thickening and mucus hypersecretion caused by urban PM_10_ (uPM_10_, particles less than 10 μm). Mice were orally administrated with 10 mg/kg aesculetin and exposed to 6 μg/mL uPM_10_ for 8 weeks. To further explore the mechanism(s) involved in inhibition of uPM_10_-induced mucus hypersecretion by aesculetin, bronchial epithelial BEAS-2B cells were treated with 1–20 µM aesculetin in the presence of 2 μg/mL uPM_10_. Oral administration of aesculetin attenuated collagen accumulation and mucus hypersecretion in the small airways inflamed by uPM_10_. In addition, aesculetin inhibited uPM_10_-evoked inflammation and oxidant production in lung tissues. Further, aesculetin accompanied the inhibition of induction of bronchial epithelial toll-like receptor 4 (TLR4) and epidermal growth factor receptor (EFGR) elevated by uPM_10_. The inhibition of TLR4 and EGFR accompanied bronchial mucus hypersecretion in the presence of uPM_10_. Oxidative stress was responsible for the epithelial induction of TLR4 and EGFR, which was disrupted by aesculetin. These results demonstrated that aesculetin ameliorated airway thickening and mucus hypersecretion by uPM_10_ inhalation by inhibiting pulmonary inflammation via oxidative stress-stimulated TLR4 and EGFR. Therefore, aesculetin may be a promising agent for treating airway mucosa-associated disorders elicited by urban coarse particulates.

## 1. Introduction

Particulate matter (PM) is a generic term to categorize air pollutants consisting of a mixture of solid and liquid particles suspended in the air, varying in composition, size, and physical features [1,2]. Industrial facilities, power plants, vehicles, incinerators, dust, and fires are the major anthropogenic sources of urban PM. The ability of PM to engender potential risk to human health is dependent on the size and chemical composition of the suspended particles [2,3]. The particle sizes range between 2.5 mm (PM_2.5_) and 10 mm (PM_10_). Airborne PM with potential toxic elements can act as a lethal blow to health and in particular as a leading driver of premature mortality and cardiopulmonary morbidity [2,4]. Potential toxic elements of PM include inorganic ions, organic substances, metals, reactive gases, and particle carbon core [5]. Especially, PM affects different parts of the respiratory tracts according to the size of particle, eventually eliciting diverse types of respiratory diseases [6,7]. The upper airways are damaged by PM_10_, whereas ultrafine particles (less than 0.1 μm) influence lung alveoli [8,9]. It is reasonable to assume that finer particles are more hazardous to human health than the coarser ones [10]. PM can cause other noxious diseases such as nonfatal heart attacks, serious asthma, reduced lung functionality, airway irritation, and difficult breathing [11,12].

There is a growing body of evidence demonstrating that the adverse effects of airborne PM on respiratory or cardiovascular diseases are linked to its deposition in the respiratory tract [4,13]. Numerous studies have investigated which mechanisms are involved in respiratory health effects in response to PM [14,15,16]. The airway epithelium, as the major site of PM deposition, may undergo barrier dysfunction as the initial effects of PM [4]. The air pollution-induced toxic effects involve the formation of reactive oxygen species (ROS) through activation of direct and indirect mechanisms [4,17,18]. Oxidative stress may lead to activation of the inflammatory cascades in both macrophages and epithelial cells [17]. In addition, oxidative stress can trigger redox-sensitive apoptotic pathways leading to oxidative organ injury [17]. However, the susceptibility of the target organ to injury may be attributed to its capability to enhance protective scavenging systems involving endogenous redox signaling and potent immune-mediated responses [17,19]. A recent study shows that the nuclear factor erythroid-related factor 2 activated by redox imbalance plays a key role in alleviating PM-induced toxicity through inhibiting oxidative damage [20]. Hydrogen sulfide, an endogenous gaseous molecule present in the circulation, attenuates PM-induced pulmonary inflammatory injury, lung endothelial barrier disruption, and vascular hyperpermeability [21]. Nevertheless, the role of antioxidant defenses to chronic exposure of PM needs further exploration.

**Figure 1:**
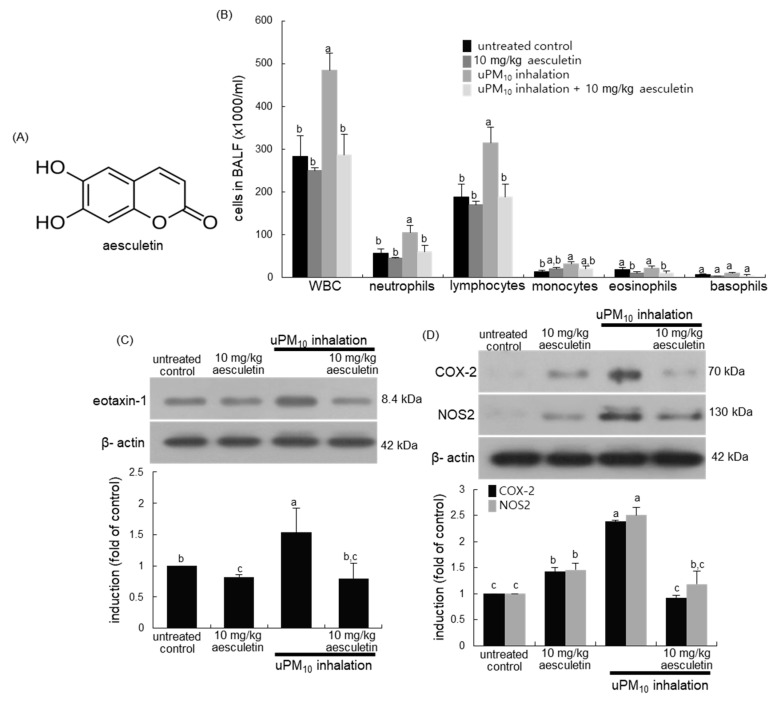
Chemical structure (**A**), leukocytes in bronchoalveolar lavage fluid (BALF, (**B**)) and induction of inflammatory COX-2 and NOS2 (**C**), and eotaxin-1 (**D**) in uPM_10_-exposed and aesculetin-treated mouse lung tissues. Mice exposed to uPM_10_ were orally treated with 10 mg/kg aesculetin. Cells in the BALF were counted by using a hematologic analyzer (**B**). Lung tissue extracts were subject to Western blot analysis. The graphs (mean ± SEM, n = 3) denote quantitative densitometric results. Respective values in the bar graphs not sharing an alphabetical lowercase imply significant difference at *p* < 0.05.

Since PM is associated with inflammatory respiratory diseases, including chronic obstructive pulmonary disease (COPD) and asthma, potential nutrients and supplements with an anti-inflammatory property have been suggested for their prevention and treatment [22,23]. For example, stemonine, a traditional Chinese herb, reduces lung inflammation in mice with PM_2.5_-induced COPD, providing a novel approach for the treatment of PM2.5-induced respiratory diseases [24]. In addition, the harmful effects of PM on respiratory diseases may be ameliorated by safe and effective natural antioxidants [24]. Experimental studies have shown that natural plant compounds have antioxidant and anti-inflammatory effects on PM-exposed target cells [25]. Aesculetin (Figure 1A), a coumarin compound present in the Sancho tree and chicory, has anti-inflammatory and anti-diabetic effects [26,27,28]. Aesculetin could be effective in protecting against acute lung injury via disruption of inflammatory pathways [28]. Our recent study reveals that aesculetin attenuates alveolar damage and fibrosis via interaction of alveolar epithelial cells with blood-derived macrophages [29]. However, little is known about the beneficial effects of aesculetin on PM-induced airway thickening and mucus hyperproduction. The current study elucidated that aesculetin ameliorated aberrant mucus hypersecretion from mouse airways exposed to urban PM_10_ (uPM_10_). In addition, this study examined whether aesculetin ameliorated airway thickening of mice exposed to uPM_10_ inhalation. Furthermore, the underlying mechanisms involved in airway thickening and mucus overproduction were explored by examining inflammatory receptor induction and oxidant formation in bronchial epithelial cells exposed to uPM_10_.

## 2. Materials and Methods

### 2.1. Chemicals

M199 culture media and human epidermal growth factor (EGF) were obtained from the Sigma-Aldrich Chemical (St. Louis, MO, USA), as were all other reagents, unless specifically stated elsewhere. Fetal bovine serum (FBS), penicillin-streptomycin, and trypsin-EDTA were offered by Lonza (Walkersville, MD, USA). Human bronchial airway epithelial cell line, BEAS-2B, was provided from the American Type Culture Collection (ATCC, Manassas, VA, USA). Sigma-Aldrich Chemical provided uPM_10_ sample (product number: NIST SRM 1648A, particles less than 10 μm). For the Western blot analysis, antibodies against toll-like receptor 4 (TLR4), cyclooxygenase-2 (COX-2), nitric oxide synthase 2 (NOS2), eotaxin-1, α-smooth muscle actin (α-SMA), and collagen I were obtained from the Santa Cruz Biotechnology (Santa Cruz, CA, USA). Human collagen IV antibody was purchased from Bioss Antibodies (Woburn, MA, USA). Antibodies of human EGF receptor (EGFR) and MUC5AC were supplied by Abcam (Cambridge, UK).

Aesculetin (Sigma-Aldrich Chemical) was prepared in dimethyl sulfoxide (DMSO, <0.5% in a final culture concentration) for cell culture.

### 2.2. Animal Experiments

Five week-old male BALB/c mice (Hallym University Breeding Center for Laboratory Animals) introduced in this study were bred at the Animal Facility of Hallym University. Female mice were excluded due to concerns that female hormone cycles would influence experimental results. Mice were acclimatized for 1 week before commencing the experiments. Mouse caring and feeding were described in our previous study [29]. The current study was approved by the Hallym University Institutional Review Board and the Committee on Animal Experimentation (Hallym 2017-56, approved on 14 February 2018) in compliance with the University’s Guidelines for the Care and Use of Laboratory Animals.

All mice were divided into four subgroups (n = 6–7 for each subgroup). Mice receiving uPM_10_ inhalation were further placed into two subgroups. Mice were supplied with 10 mg/kg aesculetin via oral gavage daily for 8 weeks, and 1 h later mice inhaled 6 μg/mL uPM_10_ for 30 min in a plastic chamber with an ultrasonic nebulizer (Clenny2 Aerosol, Medel, Italy). Control mice and aesculetin-treated mice received phosphate-buffered saline (PBS) as a vehicle of the particulate matter. All mice were sacrificed 24 h after the latest provocation (day 60) with an anesthetic [29]. The trachea, lungs and airways were cleaned in 1 mL PBS to collect bronchoalveolar lavage fluid (BALF). The amounts of inflammatory cells were measured using a hematologic analyzer (Drew Scientific, Oxford, CT, USA). The right lungs were placed in liquid nitrogen and the left lungs fixed in 4% paraformaldehyde for immunohistological analyses.

### 2.3. Western Blot Analysis

Western blot analysis was conducted with the protocols described in our previous study [29]. Lung tissue extracts and cell lysates and culture media were electrophoresed and transferred on a nitrocellulose membrane. The gel membrane was incubated overnight at 4 °C with a specific primary antibody of MUC5AC, COX-2, NOS2, eotaxin-1, α-SMA, collagen I, collagen IV, TLR4, and EGFR. The membrane was then applied to a secondary antibody conjugated to horseradish peroxidase (HRP) for 1 h. For the comparative control, β-actin antibody was used.

### 2.4. Hematoxylin and Eosin (H&E) Staining

For the histological analyses of airways, small airway and alveolar specimens provided at the end of the experiments were fixed in 10% paraformaldehyde [30]. The H&E-stained tissue sections were examined using an optical microscope Axioimager system equipped for fluorescence illumination (Carl Zeiss, Oberkochen, Germany). Three images were taken from each tissue section.

### 2.5. Masson Trichrome Staining

The sectioned paraffin-embedded lung tissues were stained with Masson trichrome stain for the visualization of collagen fibers. The sections were examined by using an optical Axiomager microscope (Carl Zeiss), and three images were taken for each section.

### 2.6. Alcian Blue-Periodic Acid-Schiff (PAS) Staining

The paraffin-embedded lung specimens were sectioned and stained with PAS stain (Sigma-Aldrich Chemical) to assess goblet cell hyperplasia as a measure of airway mucus hypersecretion [31]. The stained sections were examined, and five images were taken for each section.

### 2.7. Dihydroethidium (DHE) Staining for ROS Production

Paraffin-embedded tissue sections of airways were prepared for DHE staining [30]. Airway tissues were stained by incubating for 1 h in 20 μM DHE (Invitrogen, Carlsbad, CA, USA). For the identification of nuclei, 4′,6-diamidino-2-phenylindole (DAPI) was given for 10 min. Stained tissues on slides were mounted and images were taken using an optical microscope (Carl Zeiss).

### 2.8. BEAS-2B Cell Culture

BEAS-2B cells were cultured in 25 mM HEPES-buffered M199 containing 10% FBS, according to culture protocols previously described in our studies [31]. Aesculetin at 1–20 μM was pretreated overnight, and then 2 μg/mL uPM_10_ applied to BEAS-2B cells to induce TLR4, EGFR, MUC5AC, collagen I, and collagen IV. Peak expressions of TLR4 and EGFR were attained when uPM_10_ was added to BEAS-2B cells for 8 h and MUC5AC for 48 h.

The BEAS-2B cytotoxicity of ≤20 μM aesculetin was determined after 24 h culture by using a 3-(4,5-dimethylthiazol-yl)-diphenyl tetrazolium bromide (MTT, Duchefa Biochemie, Haarlem, The Netherlands) assay [29].

### 2.9. Statistical Analysis

The data (means ± SEM) obtained from the in vitro and in vivo experiments were run for statistical analyses [29,30,31]. Significance was determined by a one-way ANOVA and differences were analyzed with Duncan’s multiple-range test at *p* < 0.05.

## 3. Results

### 3.1. Inhibitory Effects of Aesculetin on uPM_10_-Induced Lung Inflammation

This study investigated whether uPM_10_ inhalation influenced leukocyte contents in BALF. When 6 μg/mL uPM_10_ was inhaled to mice for 8 weeks, the amounts of neutrophils and lymphocytes in BALF highly augmented (Figure 1B). In contrast, the supplement of 10 mg/kg aesculetin to uPM_10_-exposed mice inhibited neutrophilia and lymphocytosis in BALF. Further, this study examined whether uPM_10_ evoked lung inflammation was attenuated by oral administration of aesculetin. The induction of inflammatory COX-2 and NOS2 was markedly elevated in uPM_10_-inhaled lung tissues and such induction was inhibited by 10 mg/kg aesculetin (Figure 1C). In addition, aesculetin diminished airway induction of eotaxin-1 elevated in the presence of uPM_10_ (Figure 1D), indicating this compound may inhibit eosinophilia in airways.

### 3.2. Suppressive Effects of Aesculetin on Thickening of uPM_10_-Inhaled Airways

**Figure 2:**
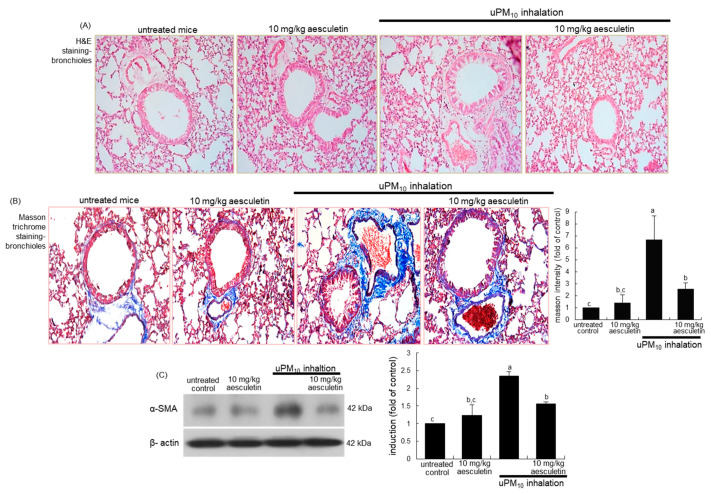
Inhibitory effects of aesculetin on airway thickening (**A**), fibrosis (**B**) and α-SMA accumulation of uPM_10_-exposed mouse small airways. Mice were orally supplied with 10 mg/kg aesculetin and exposed to uPM_10_ for 8 weeks. Airway tissues were stained by using H&E reagent (**A**) and Masson trichrome stain (**B**). Each photograph is representative of four mice. Lung tissue extracts were subjected to Western blot analysis (**C**). The bar graphs (mean ± SEM, n = 3) show quantitative results. Respective values in bar graphs not sharing an alphabetical lowercase show significant difference at *p* < 0.05.

This study examined whether aesculetin attenuated airway thickening due to uPM_10_ inhalation. From the histological examination with H&E staining, the small airways of uPM_10_-exposed mice became thick and narrow (Figure 2A). When 10 mg/kg aesculetin was supplemented to uPM_10_-loaded mice, such histological thickening in the small airways was noticeably reduced (Figure 2A). Collagen fiber deposits were notably observed around airways of uPM_10_ inhalation-challenged mice, as detected by Masson trichrome staining (Figure 2B). It should be noted that the uPM_10_ inhalation proliferated goblet cells in the small airways. However, supplying aesculetin for 8 weeks lessened the deposition of collagen fibers and goblet cell hyperplasia (Figure 2B). Consistently, the treatment of 10 mg/kg aesculetin reduced the airway tissue level of α-SMA enhanced in uPM_10_-challenged mice (Figure 2C).

### 3.3. Blockade of uPM_10_-Induced Airway Mucus Hypersecretion by Aesculetin

**Figure 3:**
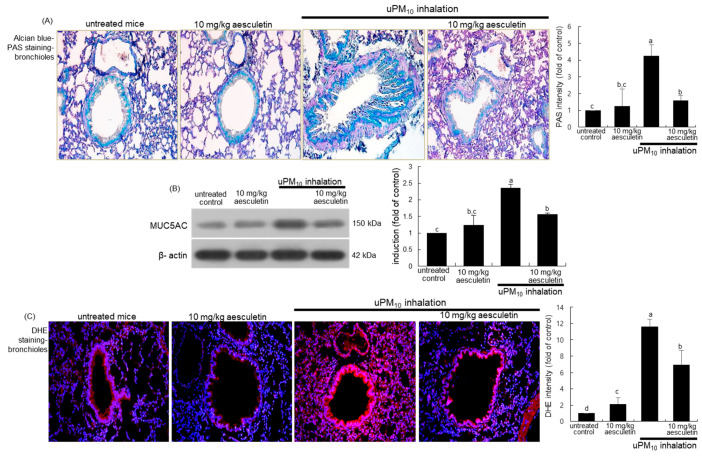
Inhibition of production of mucin (**A**,**B**) and oxidants (**C**) by 10 mg/kg aesculetin in uPM_10_-exposed mouse small airways. Mice were orally administrated with 10 mg/kg aesculetin and exposed to uPM_10_ for 8 weeks. Airway tissue sections were stained by using Alcian blue-periodic acid-schiff (PAS, (**A**)) and dihydroethidium (DHE) stains (**C**). Each photograph is representative of four mice. Nuclear counterstaining was conducted with hematoxylin (**A**) and DAPI (**C**). Lung tissue extracts were applied to Western blot analysis (**B**). The bar graphs (mean ± SEM, n = 3) indicate quantitative results. Respective values in bar graphs not sharing an alphabetical lowercase indicate significant difference at *p* < 0.05.

This study attempted to examine whether aesculetin inhibited mucus hypersecretion in airways induced by uPM_10_ inhalation. When mice were exposed to uPM_10_ for 8 weeks, there was a strong Alcian blue-PAS staining observed in mouse airways, indicating mucus hypersecretion from epithelial goblet cells (Figure 3A). In contrast, oral administration of 10 mg/kg aesculetin markedly reduced the staining in the airway epithelium of uPM_10_ inhalation-challenged mice. Further, Western blot analysis showed that the aesculetin treatment reduced the elevated MUC5AC induction in uPM_10_-inhaled mouse small airways (Figure 3B). On the other hand, this study introduced DHE staining for the measurement of ROS production in lungs exposed to uPM_10_. A strong red DHE staining was detected in uPM_10_-loaded airways, indicating marked ROS production by uPM_10_ (Figure 3C). However, aesculetin highly reduced the ROS production in lungs. Accordingly, aesculetin inhibited the uPM_10_ inhalation-induced airway thickening and mucus overproduction possibly through diminishing oxidative stress in the airways.

### 3.4. Inhibition of uPM_10_ Induction of Epithelial Mucin and Collagen by Aesculetin

**Figure 4:**
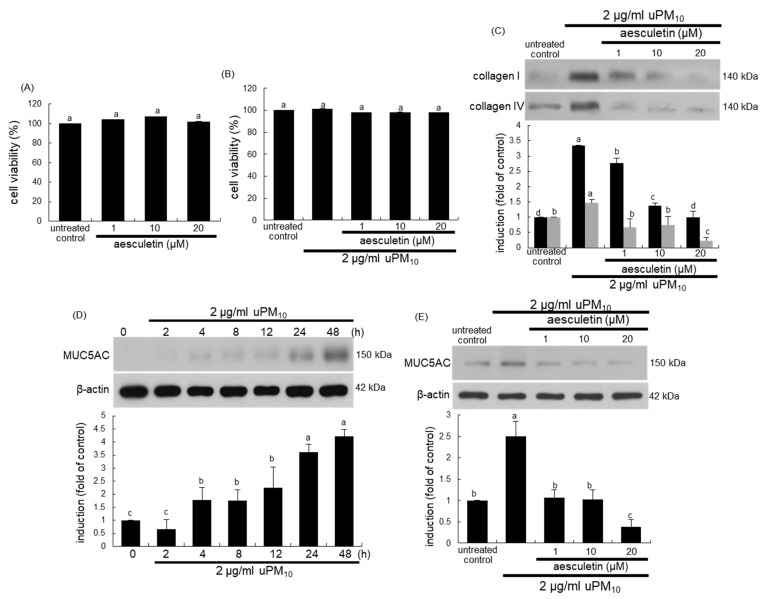
Cytotoxicity (**A**,**B**), and induction of collagens (**C**) and MUC5AC (**D**,**E**) in uPM_10_-loaded bronchial epithelial cells. BEAS-2B cells were treated with 2 μg/mL uPM_10_ in the presence of 1–20 µM aesculetin for 24 h. Cell viability was measured by MTT assay (cell viability = 100%, mean ± SEM, n = 5). Lung tissue extracts were subjected to Western blot analysis with a primary antibody against collagen I, collagen IV (**C**) and MUC5AC (**D**,**E**). The bar graphs (mean ± SEM, n = 3) designate quantitative results. β-Actin was used as an internal control. Respective values in bar graphs not sharing an alphabetical lowercase indicate significant difference at *p* < 0.05.

The current study attempted to confirm that aesculetin inhibited bronchial epithelial thickening and mucus hypersecretion induced by uPM_10_. When BEAS-2B cells were cultured with aseculetin, there was no cytotoxic effect of aesculetin in the absence and presence of 2 μg/mL uPM_10_ (Figure 4A,B). The uPM_10_ stimulation highly enhanced the cellular levels of fibrotic collagen proteins (Figure 4C). The induction of these proteins was attenuated by treating ≥1 μM aesculetin. In addition, the mucin protein MUC5AC as a mucus hypersecretion marker was highly induced in bronchial epithelial BEAS-2B cells by stimulation with uPM_10_ for 48 h (Figure 4D). In contrast, such induction was abolished by treating ≥1 μM aesculetin (Figure 4E). Accordingly, aesculetin may encumber bronchial epithelial thickening and mucus hyperproduction triggered by uPM_10_.

### 3.5. Suppression of uPM_10_-Induced Inflammatory Epithelial Receptors by Aesculetin

**Figure 5:**
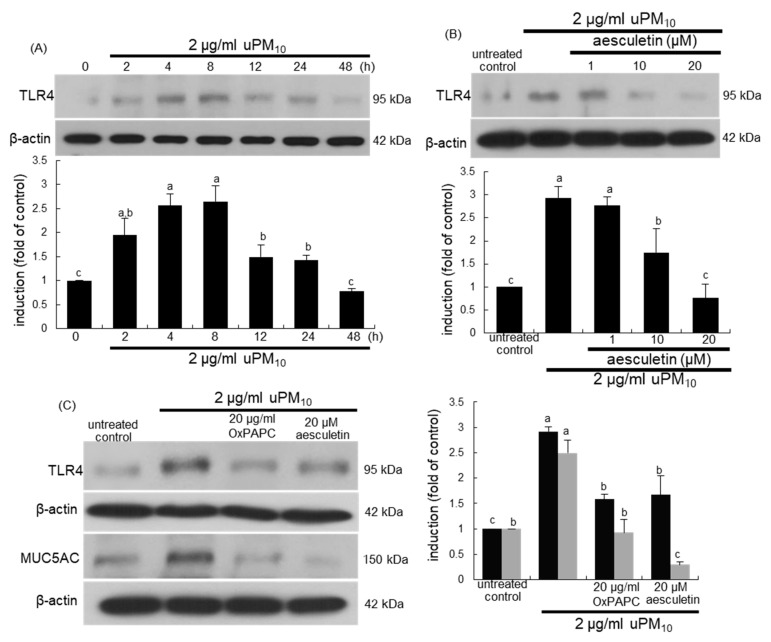
Western blot analysis showing temporal responses of TLR4 induction (**A**), and TLR4 inhibition by aesculetin (**B**) and MUC5AC blockade by TLR4 inhibitor (**C**) in uPM_10_-exposed bronchial epithelial cells. BEAS-2B cells were incubated with 2 μg/mL uPM_10_ up to 48 h in the presence of 1–20 μM aesculetin (**B**) or 20 μg/mL OxPAPC (**C**). Cell lysates were applied to Western blot analysis. The bar graphs (mean ± SEM, n = 4) designate quantitative results. Means without a common alphabetical lowercase differ, *p* < 0.05.

This study investigated that airway thickening and mucus hypersecretion due to uPM_10_ entailed the induction of airway epithelial receptors of TLR4 and EGFR. There was very weak expression of TLR4 in untreated control cells, whereas this receptor was greatly induced by treating 2 μg/mL uPM_10_ to BEAS-2B cells for 4–8 h (Figure 5A). The addition of ≥10 μM aesculetin to epithelial cells inhibited such induction of TLR4 significantly (Figure 5B). When the cells exposed to uPM_10_ in the presence of 20 μg/mL OxPAPC, a TLR2/4 signaling inhibitor, the uPM_10_-up-regulated MUC5AC induction was dampened by non-toxic OxPAPC (Figure 5C).

**Figure 6:**
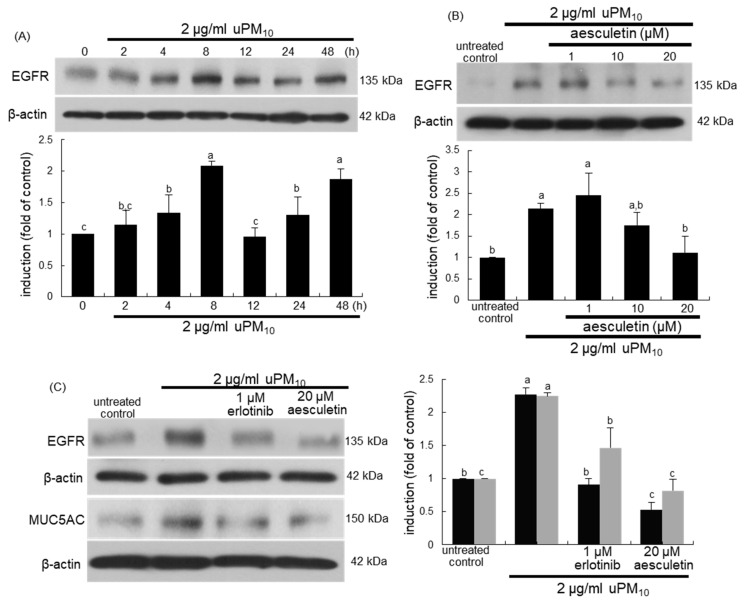
Temporal induction of epidermal growth factor receptor (EGFR) (**A**), its blockade by aesculetin (**B**) and MUC5AC inhibition by EGFR inhibitor (**C**) in uPM_10_-exposed bronchial epithelial cells. BEAS-2B cells were incubated with 2 μg/mL uPM_10_ up to 48 h in the presence of 1-20 μM aesculetin (**B**) or 1 μM erlotinib (**C**). Cell lysates were applied to Western blot analysis. The bar graphs (mean ± SEM, n = 4) show quantitative results. Means without a common alphabetical lowercase differ, *p* < 0.05.

When bronchial epithelial cells were exposed to uPM_10_, the EGFR induction showed a temporal dual-peak response, achieving peak induction at 8 h and 48 h after its exposure (Figure 6A). The treatment of epithelial cells with ≥10 μM aesculetin diminished such induction of EGFR significantly (Figure 6B). The EGFR inhibition by 1 μM erlotinib abolished epithelial induction of MUC5AC up-regulated by uPM_10_ (Figure 6C). It should be noted that more potent inhibition of the MUC5AC induction was observed with 20 μM aesculetin than with OxPAPC or erlotinib (Figure 5C). Therefore, the mucus overproduction by uPM_10_ may be attributed to simultaneous activation of both TLR4 and EGFR.

### 3.6. Involvement of Oxidative Stress in Epithelial Receptor Activation by uPM_10_

**Figure 7:**
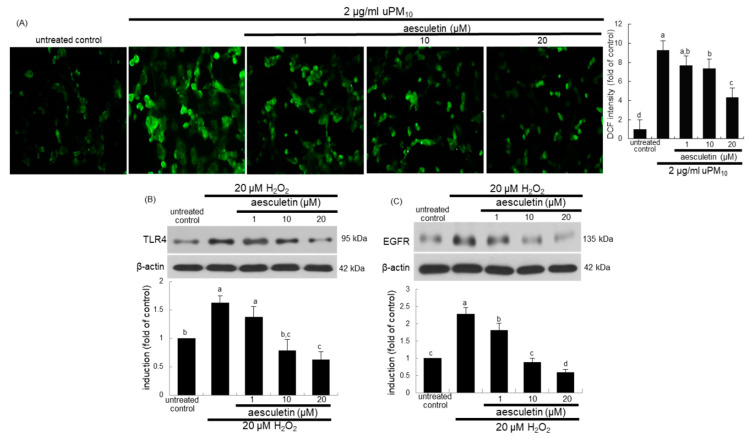
Inhibition of reactive oxygen species (ROS) production (**A**) and induction of TLR4 (**B**) and EGFR (**C**) by aesculetin in uPM_10_-exposed or H_2_O_2_-stimulated bronchial epithelial cells. BEAS-2B cells were incubated with 2 μg/mL uPM_10_ or 20 μM H_2_O_2_ for 8 h in the presence of 1–20 μM aesculetin (**B**). The ROS production was measured by fluorescent DCF-DA staining (n = 3, (**A**)). Cell lysates were applied to Western blot analysis. The bar graphs (mean ± SEM, n = 4) in the bottom panels show quantitative results. Means without a common alphabetical lowercase differ, *p* < 0.05.

This study attempted to determine that intracellular oxidant formation was involved in concurrent activation of TLR4 and EGFR in uPM_10_-exposed cells, as evidenced by oxidation of DCF-DA. As expected, a weak DCF staining was detected in non-stimulated control cells (Figure 7A). The uPM_10_-alone-exposed cells showed heavy fluorescence, indicative of marked oxidant generation. The cells exposed to uPM_10_ in the presence of 1–20 μM aesculetin showed no increase in DCF fluorescence (Figure 7A). It can be assumed that aesculetin may block an accumulation of intracellular oxidants in epithelial cells due to uPM_10_.

To examine whether oxidative stress was involved in induction of TLR4 and EGFR of uPM_10_-inhaled airway epithelial cells, BEAS-2B cells were treated with 20 μM H_2_O_2_. Treating with 20 μM H_2_O_2_ for 8 h elevated epithelial TLR4 induction (Figure 7B). Similarly, the oxidant loading induced EGFR in epithelial cells (Figure 7C). Submicromolar aesculetin attenuated such induction of TLR4 and EGFR in a dose-dependent manner (Figure 7B,C). These data imply that oxidants may trigger the receptor signaling of TLR4 and EGFR responsible for mucus overproduction due to uPM_10_.

## 4. Discussion

Epidemiological evidence supports that increased levels of particulate air pollution enhance morbidity and mortality of respiratory diseases [32,33]. There is a strong relationship between PM and exacerbations of pre-existing lung conditions such as COPD observed [34]. However, little is known about how a chronic contact to ambient PM can evoke the development of COPD. The PM deposition in the respiratory tracts is dependent predominantly on the particle size, with larger particles accumulated in the upper and larger airways, and smaller particles infiltrating deep into the alveolar spaces [8,9,34]. This study found that the exposure of mice to uPM_10_ induced bronchiolar thickening along with increased expression of α-SMA in lung tissues. In addition, there was mucus overproduction in bronchioles exposed to uPM_10_, being concurrent with increased induction of epithelial MUC5AC in airways. However, it should be noted that uPM_10_ did not show notable alveolar injury (data not shown), indicating that the particle size of uPM_10_ was not small enough to penetrate deep into the alveoli. Consistently, the uPM_10_ loading enhanced the induction of collagens and MUC5AC in bronchial airway epithelial cells. Ineffective clearance of this uPM_10_ from the bronchial airways could cause particle retention in upper and larger lung tissues. Eventually, such particle retention in airways may result in pathogenetical responses critical for respiratory diseases such as allergic asthma accompanying eosinophilia. Indeed, the uPM_10_ inhalation enhanced the eotaxin-1 induction in lung tissues.

Long-term exposure to urban air pollution leads to reduction of lung functionality, airway irritation, breathing difficulty, and aggravation of pre-existing respiratory diseases [7,12,34]. Although the precise mechanisms underlying detrimental effects of air pollutants are still unclear, the induction of pulmonary inflammation following particle inhalation represents a plausible mechanistic pathway. The airway epithelium as a major site of PM deposition is known to be inflammogenic [4]. Several studies have shown that PM retention in airways triggers pulmonary inflammation [14,15,35]. This study revealed that uPM_10_ inhalation to mice enhanced lung tissue levels of the inflammatory COX-2 and NOS2, suggesting that uPM_10_ caused pulmonary inflammation. One can assume that the mucus overproduction from uPM_10_-stimulated bronchioles may be attributed to pulmonary inflammation. According to current literature, PM generates ROS, inducing antioxidant and inflammatory responses in airway epithelial cells [4,17,19]. It was found that the exposure to uPM_10_ instigated oxidative stress in mouse bronchioles, evidenced by DHE staining. Therefore, the mucus overproduction and bronchiole thickening by uPM_10_ may entail oxidative stress leading to pulmonary inflammation. Oxidative stress as a consequence of air pollution results in organ injury via redox-sensitive apoptotic pathways [17]. Fine PM initiates type 2 cytokines-evoked pro-inflammatory immune responses in chronically inflamed upper airway mucosa [36]. However, the mechanisms underlying the unfavorable effects of uPM_10_ on mucosal diseases remain debatable.

It has been generally accepted that the pathological mechanisms through which PM may contribute to respiratory diseases include oxidative stress-triggered direct toxic injury [15,16,19,37]. Accordingly, literature evidence suggests that dietary antioxidants and supplements abrogate harmful effects of air pollution in asthma and other chronic respiratory diseases [22,23]. This study found that plant-derived aesculetin attenuated uPM_10_-induced oxidative stress and inflammation, accompanying reduction of neutrophilia and lymphocytosis in BALF. Antioxidants may alleviate the adverse effects of PM on chronic diseases [19,24,25]. Aesculetin attenuates alveolar injury and fibrosis induced by macrophage-derived inflammation [29]. Similarly, phenolic compounds may have a favorable effect on skin exposed to heavy air pollution through exerting antioxidant and anti-inflammatory activities [25]. These compounds reduce cellular ROS level, and augment cellular antioxidant ability and lower the levels of cellular mediators involved in inflammatory responses to PM [25]. Nevertheless, it is still undefined whether PM-triggered oxidative stress is involved in airway thickening and mucin overproduction. This study demonstrated that plant-derived aesculetin attenuated uPM_10_-induced airway thickening and bronchiolar mucus hyperproduction possibly through its inhibition of oxidative stress and inflammation. One study shows that naringin blocks diesel PM-induced abnormal secretion of airway surface liquid in the respiratory tract [38]. These findings suggest that plant-derived natural compounds may provide a novel strategy for the therapeutically treatment of PM-induced respiratory diseases.

Molecular scenes of the respiratory diseases associated with exposure to PM include induction of inflammation, oxidative stress, and cell death [15,16,37]. Both TLR2 and TLR4 are involved in inflammatory immune responses of macrophages induced by fine and coarse ambient air PM [39]. Urban PM_2.5_ containing trace microbial elements may exacerbate allergic inflammation and eosinophilia in the murine lung via a TLR2/TLR4/MyD88-signaling pathway [40]. This study revealed that the uPM_10_ inhalation stimulated mucus production aberrantly from airway epithelial cells overexpressing TLR4, which was abrogated by aesculetin, as the inhibitor of TLR4. On the other hand, lung inflammation by neutrophil elastase entails the EGFR activation in bronchial epithelial by a novel metalloprotease pathway [41]. The EGFR inhibition reduces both cytokine expression and endotoxicity in response to lipopolysaccharide in mice [41]. In the present study EGFR activation was involved in the mucus production from airway epithelial cells exposed to uPM_10_. The current findings suggest that coarse uPM-induced epithelial activation of both EGFR and TLR4 may influence airway inflammation via oxidative stress. Therefore, aesculetin targeting TLR4 and EGFR may find use in treating airborne PM-associated respiratory diseases. Similarly, naringenin reduces mucous hypersecretion in neutrophil elastase-induced airway inflammation by encumbering ROS production and inhibiting the NF-κB activity via EGFR-PI3K-Akt/ERK signaling [42].

## 5. Conclusions

The current report revealed that aesculetin attenuated airway thickening and mucus hypersecretion during pulmonary inflammation by uPM_10_, inducing oxidative stress concurrently. In addition, aesculetin accompanied inhibition of cellular induction of bronchial epithelial TLR4 and EFGR by stimulation with uPM_10_. Furthermore, such protective effects of aesculetin on mucus hypersecretion were attributed to the inhibition of bronchiolar epithelial induction of TLR4 and EGFR. Taken together, naturally-occurring aesculetin ameliorated upper airway mucosa inflamed by urban coarse PM inhalation by disrupting pulmonary inflammation and oxidative stress via TLR4 and EGFR.

## Data Availability

All the data presented in this study are included in the article.

## References

[1] Falcon-Rodriguez C.I., Osornio-Vargas A.R., Sada-Ovalle I., Segura-Medina P. (2016). Aeroparticles, composition, and lung diseases. Front. Immunol..

[2] Ali M.U., Liu G., Yousaf B., Ullah H., Abbas Q., Munir M.A.M. (2019). A systematic review on global pollution status of particulate matter-associated potential toxic elements and health perspectives in urban environment. Environ. Geochem. Health.

[3] Gautam S., Yadav A., Tsai C.J., Kumar P. (2016). A review on recent progress in observations, sources, classification and regulations of PM 2.5 in Asian environments. Environ. Sci. Pollut. Res. Int..

[4] Cooper D.M., Loxham M. (2019). Particulate matter and the airway epithelium: The special case of the underground?. Eur. Respir. Rev..

[5] Cassee F.R., Héroux M.E., Gerlofs-Nijland M.E., Kelly F.J. (2013). Particulate matter beyond mass: Recent health evidence on the role of fractions, chemical constituents and sources of emission. Inhal. Toxicol..

[6] Fiordelisi A., Piscitelli P., Trimarco B., Coscioni E., Iaccarino G., Sorriento D. (2017). The mechanisms of air pollution and particulate matter in cardiovascular diseases. Heart Fail. Rev..

[7] Losacco C., Perillo A. (2018). Particulate matter air pollution and respiratory impact on humans and animals. Environ. Sci. Pollut. Res. Int..

[8] Peters A., Veronesi B., Calderón-Garcidueñas L., Gehr P., Chen L.C., Geiser M., Reed W., Rothen-Rutishauser B., Schürch S., Schulz H. (2006). Translocation and potential neurological effects of fine and ultrafine particles a critical update. Part. Fibre Toxicol..

[9] Suhaimi N.F., Jalaludin J. (2015). Biomarker as a research tool in linking exposure to air particles and respiratory health. BioMed Res. Int..

[10] Korhonen A., Lehtomäki H., Rumrich I., Karvosenoja N., Paunu V.V., Kupiainen K., Sofiev M., Palamarchuk Y., Kukkonen J., Kangas L. (2019). Influence of spatial resolution on population PM2.5 exposure and health impacts. Air Qual. Atmos. Health.

[11] Kim H.J., Choi M.G., Park M.K., Seo Y.R. (2017). Predictive and prognostic biomarkers of respiratory diseases due to particulate matter exposure. J. Cancer Prev..

[12] Yang I.A., Fong K.M., Zimmerman P.V., Holgate S.T., Holloway J.W. (2008). Genetic susceptibility to the respiratory effects of air pollution. Thorax.

[13] Kecorius S., Madueño L., Löndahl J., Vallar E., Galvez M.C., Idolor L.F., Gonzaga-Cayetano M., Müller T., Birmili W., Wiedensohler A. (2019). Respiratory tract deposition of inhaled roadside ultrafine refractory particles in a polluted megacity of South-East Asia. Sci. Total Environ..

[14] Buonfiglio L.G.V., Comellas A.P. (2020). Mechanism of ambient particulate matter and respiratory infections. J. Thorac. Dis..

[15] Leikauf G.D., Kim S.H., Jang A.S. (2020). Mechanisms of ultrafine particle-induced respiratory health effects. Exp. Mol. Med..

[16] MacNee W., Donaldson K. (2003). Mechanism of lung injury caused by PM_10_ and ultrafine particles with special reference to COPD. Eur. Respir. J..

[17] Lodovici M., Bigagli E. (2011). Oxidative stress and air pollution exposure. J. Toxicol..

[18] Jin S.P., Li Z., Choi E.K., Lee S., Kim Y.K., Seo E.Y., Chung J.H., Cho S. (2018). Urban particulate matter in air pollution penetrates into the barrier-disrupted skin and produces ROS-dependent cutaneous inflammatory response in vivo. J. Dermatol. Sci..

[19] Rao X., Zhong J., Brook R.D., Rajagopalan S. (2018). Effect of particulate matter air pollution on cardiovascular oxidative stress pathways. Antioxid. Redox Signal..

[20] Pardo M., Qiu X., Zimmermann R., Rudich Y. (2020). Particulate matter toxicity is NRF2 and mitochondria dependent: The roles of metals and polycyclic aromatic hydrocarbons. Chem. Res. Toxicol..

[21] Wang T., Wang L., Zaidi S.R., Sammani S., Siegler J., Moreno-Vinasco L., Mathew B., Natarajan V., Garcia J.G.N. (2012). Hydrogen sulfide attenuates particulate matter-induced human lung endothelial barrier disruption via combined reactive oxygen species scavenging and Akt activation. Am. J. Respir. Cell. Mol. Biol..

[22] Whyand T., Hurst J.R., Beckles M., Caplin M.E. (2018). Pollution and respiratory disease: Can diet or supplements help? A review. Respir. Res..

[23] Romieu I., Castro-Giner F., Kunzli N., Sunyer J. (2008). Air pollution, oxidative stress and dietary supplementation: A review. Eur. Respir. J..

[24] Zhang J., Li S., Sun L., Chen Y., Zhang L., Zhang Z. (2017). Therapeutic effects of stemonine on particulate matter 2.5-induced chronic obstructive pulmonary disease in mice. Exp. Ther. Med..

[25] Boo Y.C. (2019). Can plant phenolic compounds protect the skin from airborne particulate matter?. Antioxidants.

[26] Kang K.S., Lee W., Jung Y., Lee J.H., Lee S., Eom D.W., Jeon Y., Yoo H.H., Jin M.J., Song K.I. (2014). Protective effect of esculin on streptozotocin-induced diabetic renal damage in mice. J. Agric. Food Chem..

[27] Witaicenis A., Seito L.N., Di Stasi L.C. (2010). Intestinal anti-inflammatory activity of esculetin and 4-methylesculetin in the trinitrobenzenesulphonic acid model of rat colitis. Chem. Biol. Interact..

[28] Lee H.C., Liu F.C., Tsai C.N., Chou A.H., Liao C.C., Yu H.P. (2020). Esculetin ameliorates lipopolysaccharide-induced acute lung injury in mice via modulation of the AKT/ERK/NF-κB and RORγt/IL-17 pathways. Inflammation.

[29] Oh S.Y., Kim Y.H., Kang M.K., Lee E.J., Kim D.Y., Oh H., Kim S.I., Na W., Kang Y.H. (2020). Aesculetin attenuates alveolar injury and fibrosis induced by close contact of alveolar epithelial cells with blood-derived macrophages via il-8 signaling. Int. J. Mol. Sci..

[30] Kim Y.H., Kang M.K., Lee E.J., Kim D.Y., Oh H., Kim S.I., Oh S.Y., Kim K.H., Park S.J., Choi Y.J. (2019). Dried yeast extracts curtails pulmonary oxidative stress, inflammation and tissue destruction in a model of experimental emphysema. Antioxidants.

[31] Kim Y.H., Choi Y.J., Lee E.J., Kang M.K., Park S.H., Kim D.Y., Oh H., Park S.J., Kang Y.H. (2017). Novel glutathione-containing dry-yeast extracts inhibit eosinophilia and mucus overproduction in a murine model of asthma. Nutr. Res. Pract..

[32] Pope C.A., Burnett R.T., Thun M.J., Calle E.E., Krewski D., Ito K., Thurston G.D. (2002). Lung cancer, cardiopulmonary mortality, and long-term exposure to fine particulate air pollution. JAMA.

[33] Brunekreef B., Forsberg B. (2005). Epidemiological evidence of effects of coarse airborne particles on health. Eur. Respir. J..

[34] Ling S.H., van Eeden S.F. (2009). Particulate matter air pollution exposure: Role in the development and exacerbation of chronic obstructive pulmonary disease. Int. J. Chron. Obstruct. Pulmon. Dis..

[35] Chan Y.L., Wang B., Chen H., Ho K.F., Cao J., Hai G., Jalaludin B., Herbert C., Thomas P.S., Saad S. (2019). Pulmonary inflammation induced by low-dose particulate matter exposure in mice. Am. J. Physiol. Lung Cell. Mol. Physiol..

[36] Qing H., Wang X., Zhang N., Zheng K., Du K., Zheng M., Li Y., Chang Y., Zhang L., Bachert C. (2019). The effect of fine particulate matter on the inflammatory responses in human upper airway mucosa. Am. J. Respir. Crit. Care Med..

[37] Traboulsi H., Guerrina N., Iu M., Maysinger D., Ariya P., Baglole C.J. (2017). Inhaled pollutants: The molecular scene behind respiratory and systemic diseases associated with ultrafine particulate matter. Int. J. Mol. Sci..

[38] Shi R., Su W.W., Zhu Z.T., Guan M.Y., Cheng K.L., Fan W.Y., Wei G.Y., Li P.B., Yang Z.Y., Yao H.L. (2019). Regulation effects of naringin on diesel particulate matter-induced abnormal airway surface liquid secretion. Phytomedicine.

[39] Shoenfelt J., Mitkus R.J., Zeisler R., Spatz R.O., Powell J., Fenton M.J., Squibb K.A., Medvedev A.E. (2009). Involvement of TLR2 and TLR4 in inflammatory immune responses induced by fine and coarse ambient air particulate matter. J. Leukoc. Biol..

[40] He M., Ichinose T., Yoshida Y., Arashidani K., Yoshida S., Takano H., Sun G., Shibamoto T. (2017). Urban PM_2.5_ exacerbates allergic inflammation in the murine lung via a TLR2/TLR4/MyD88-signaling pathway. Sci. Rep..

[41] Bergin D.A., Greene C.M., Sterchi E.E., Kenna C., Geraghty P., Belaaouaj A., Taggart C.C., O’Neill S.J., McElvaney N.G. (2008). Activation of the epidermal growth factor receptor (EGFR) by a novel metalloprotease pathway. J. Biol. Chem..

[42] Yang J., Li Q., Zhou X.D., Kolosov V.P., Perelman J.M. (2011). Naringenin attenuates mucous hypersecretion by modulating reactive oxygen species production and inhibiting NF-κB activity via EGFR-PI3K-Akt/ERK MAPKinase signaling in human airway epithelial cells. Mol. Cell. Biochem..

